# The Effect of *Lactobacillus plantarum 299v* on the Incidence of *Clostridium difficile* Infection in High Risk Patients Treated with Antibiotics

**DOI:** 10.3390/nu7125526

**Published:** 2015-12-04

**Authors:** Agata Kujawa-Szewieczek, Marcin Adamczak, Katarzyna Kwiecień, Sylwia Dudzicz, Magdalena Gazda, Andrzej Więcek

**Affiliations:** Department of Nephrology, Transplantation and Internal Medicine, Medical University of Silesia in Katowice, School of Medicine in Katowice, Katowice 40-027, Poland; agata.szewieczek@gmail.com (A.K.-S.); madamczak1@op.pl (M.A.); kapril@go2.pl (K.K.); sylwia.dudzicz@gmail.com (S.D.); m.gazda88@gmail.com (M.G.)

**Keywords:** *Lactobacillus plantarum 299v*, *Clostridium difficile*, kidney transplantation, kidney and pancreas transplantation, liver transplantation, nephrology and transplantation ward

## Abstract

**Background:** *Lactobacillus plantarum 299v* (LP299v) has been used in order to reduce gastrointestinal symptoms during antibiotic exposure. However, it remains controversial whether or not probiotics are effective in the prevention of *Clostridium difficile* infections (CDI) among patients receiving antibiotics. The aim of this study was to analyze the CDI among patients receiving antibiotics and hospitalized in the period before and after starting routine use of LP299v as a prevention of this infection. **Methods:** Among 3533 patients hospitalized in the nephrology and transplantation ward during a two-year period, 23 patients with CDI were diagnosed and enrolled in this retrospective study. Since November 2013, prevention of CDI with oral use of LP299v was performed in all patients treated with antibiotics and who were at a high risk of developing CDI. The observation period was divided into two twelve-month intervals before and after initiation of the use of LP299v as a prophylactic against CDI. Results: A significant (*p* = 0.0001) reduction of the number of cases of CDI was found after routinely using LP299v (*n* = 2; 0.11% of all hospitalized patients) compared with the previous twelve-month period of observation (*n* = 21; 1.21% of all hospitalized patients). **Conclusions:** Routine use of LP299v during treatment with antibiotics may prevent *C. difficile* infection in the nephrology and transplantation ward.

## 1. Introduction

*Clostridium difficile* is currently one of the most common identifiable pathogens of antibiotic-associated diarrhea [1,2,3] and also a major cause of nosocomial diarrhea [4]. A significant increase in the incidence and the mortality due to *C. difficile* infection has been observed during the last two decades [5,6]. Moreover, the higher rate of severe, complicated infection, treatment failure and recurrences has also been reported [7,8]. Finally, it is well documented that patients after a solid organ transplantation and receiving immunosuppressive therapy for any other reason are particularly prone to *C. difficile* infection [9,10,11].

So far, it remains controversial whether probiotics are effective in the prevention of *Clostridium difficile* infections (CDI) among patients receiving antibiotics. In a meta-analysis of 23 randomised controlled trials (*n* = 4213) Goldenberg *et al.* found that probiotics reduce the risk of *Clostridium difficile* associated diarrhea by 64% (RR 0.36; 95% CI 0.26 to 0.51). However, the subanalysis of 13 trials evaluating the effect of probiotics in reducing the incidence of CDI did not reveal a statistically significant effect of such a therapy [12]. Allen *et al.* in the largest up to date clinical study PLACIDE (supported with systematic review) evaluating the role of probiotics in the prevention of antibiotic-associated diarrhea and *Clostridium difficile* diarrhea in older inpatients (>65 years old), identified no evidence that a multistrain preparation of lactobacilli and bifidobacteria was effective in the prevention of antibiotic- associated diarrhea or *C. difficile* diarrhea. The systematic review did not include the study evaluating efficacy of the LP299v strain in antibiotic- associated diarrhea prevention [13]. However, it has been argued that, although the sample size of PLACIDE was very large, the event rate of antibiotic- associated diarrhea and CDI was much lower than predicted [14]. 

The strain 299v of *Lactobacillus plantarum* (LP299v) is a Gram-positive lactic acid bacteria, that was isolated from the mucosa of the human intestines and is also commonly found in food products [15]. It has been reported, that LP299v was isolated from faeces and from rectal and jejunal biopsies even after several days from the end of oral administration of this strain [16]. Specific properties of the colonization in the gut mucosa is probably due to mannose-dependent adhesion of LP299v to the epithelium of human intestines [17,18]. Other adhesins (glyceraldehyde-3-phosphate dehydrogenase—GAPDH), enolase—ENO and phosphoglycerate kinase—PGK) on the LP299V surface can prevent adhesion of many pathogens to the intestinal epithelium [19]. This mechanism seems to be crucial regarding the ability of LP299v to decrease bacterial translocation [20,21,22,23] and to modulate the inflammatory response of the epithelium [24]. Moreover, it has been reported the antimicrobial activity of LP299v [25,26] and several lines of evidence exist which may suggest that this strain can inhibit pathogen adhesion [27,28]. *Lactobacillus plantarum 299v* has been found to reduce clinical symptoms of irritable bowel syndrome [29,30] and gastrointerstinal adverse events during antibiotic exposure [31]. However, limited data are available regarding the efficacy of LP299v for preventing *Clostridium difficile* infections [32,33,34].

The aim of this retrospective study was to analyze the *C. difficile* infections among hospitalized patients receiving antibiotics in the period before and after initiation of routine use of LP299v, as a prevention of CDI, in the nephrology and transplantation ward.

## 2. Material and Methods

Among 3533 patients hospitalized in the Department of Nephrology, Transplantation and Internal Medicine, Medical University of Silesia in Katowice during a two-year period (October 2012–October 2013 and December 2013–December 2014), 23 patients with CDI were diagnosed and enrolled into this retrospective, observational single-centre study. As a routine, clinical practice in our Department all patients treated with antibiotics were given probiotics as a prevention of antibiotic-associated diarrhoea or *C. difficile* infection. In the period between October 2012 and October 2013, the most frequently used probiotics were: *Saccharomyces boulardii*, *Lactobacillus rhamnosus*, *Lactobacillus acidophilus*, *Lactobacillus delbrueckii* and *Bifidobacterium lactis*. Since November 2013, the routine use of LP299v as a prevention of *C. difficile* infection was introduced in all patients treated with antibiotics and at high risk of CDI developing (patients after organ transplantation and receiving immunosuppressive therapy for any other reason). For further analysis, the observation period was divided into two twelve-month intervals before (October 2012 to October 2013) and after initiation of the use of LP299v (December 2013 to December 2014). Since November 2013, all patients after organ transplantation or receiving immunosuppressive drugs for any other reason were given one capsule of LP299v orally per day (Sanprobi IBS^®^ International Pharmaceutical Consulting Sp. z o.o., Sp. k., Szczecin, Poland; capsules manufacturer—Institute Rosell-Lallemand, Montreal, Canada; LP299v strain owner—Probi AB, Lund, Sweden) during the entire period of antibiotic therapy. According to the information provided by the manufacturer, one capsule of Sanprobi IBS^®^ contains at least 10 × 10^9^ colony forming units (CFU) of LP299v.

The CDI definition was based on the current recommendations of the European Society of Clinical Microbiology and Infectious Diseases (ESCMID) [35] and was diagnosed in a two-step algorithm. The enzyme immunoassay Premier^®^ Toxins A&B test (Meridian Bioscience, Inc., Cincinnati, OH, United States) was performed for *C. difficile* toxin detection in stool samples obtained from patients with diarrhea. All stool samples with initial negative toxin assay results were evaluated subsequently using the toxigenic culture with chromID^®^ *C. difficile* culture media (bioMérieux S.A., Marcy L’Etoile, France) under anaerobic conditions. After 48 h, organisms cultured on selective media (samples) were tested for toxin production once again with enzyme immunoassay Premier^®^ Toxins A&B test (Meridian Bioscience, Inc., Cincinnati, OH, United States).

Recurrent CDI was defined as the recurrence of the infection within two months after the resolution of the previous episode. Severe CDI was diagnosed in the following cases: hospitalization in case of community-acquired CDI, presence of *megacolon toxicum* or perforation which may necessitate colectomy, admission to the intensive care unit or death secondary to CDI within 30 days from onset of the symptoms.

Nosocomial infection was defined as those occurring after 48 h of hospital admission. Community-acquired CDI was diagnosed in case of onset of symptoms up to 48 h of hospital admission or before the admission, if the patient had healthcare exposure within more than the last three months.

The following risk factors of severe CDI were included in the analysis: age >70 years, ileus, CDI suggested computer tomographic findings, elevated white blood cell count >20 G/L, serum creatinine concentration >2 mg/dL, serum albumin concentration <2.5 g/L and previous surgery within the last 30 days.

Statistical analysis was performed using the STATISTICA 7.0 PL for Windows software package (StatSoft Polska, Kraków, Poland) and MedCalc 11.3.8. (Mariakerke, Gent, Belgium). The results are presented as mean values and standard deviations. Statistical significance of between-group differences was evaluated by analysis of chi^2^ test. In all statistical tests the “*p*” values below 0.05 were considered statistically significant.

## 3. Results

The characteristics of the study group are shown in Table 1.

**Table 1:** Characteristics of patients diagnosed with *Clostridium difficile* infections (CDI) (*n* = 23).

	Before LP299v Use (*n* = 21)	After LP299v Use (*n* = 2)
Age (years)	57 ± 15	53 ± 16
Sex (M/F)	11/10	2/0
BMI (kg/m^2^)	24.0 ± 4.5	23.0 ± 0.0
Diabetes (n/%)	(6/29)	(2/100)
Chronic kidney disease (*n*/%)	(16/76)	[1/50]
Dialysis (*n*/%)	(3/14)	(0)
Systemic vasculitis (*n*/%)	(3/14)	(0)
Liver cirrhosis (*n*/%)	(3/14)	(1/50)
Inflammatory bowel disease (*n*/%)	(0)	(0)
Cancer (*n*/%)	(4/19)	(0)
Patients after organ transplantation (*n*/%)	(10/48)	(2/100)

**Figure 1:**
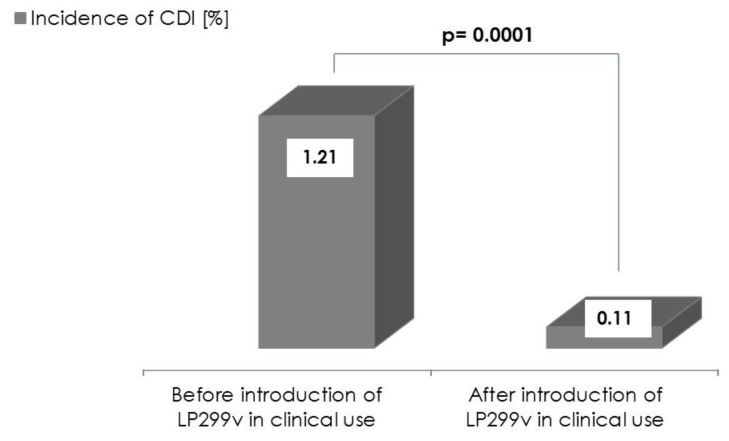
The incidence of CDI before and after administration of *Lactobacillus plantarum 299v* (LP299v).

The year before the introduction of LP299v (between October 2012 and October 2013), CDI was diagnosed in 21 patients. After initiation of the LP299v prophylaxis, in the period between December 2013 and December 2014, only two cases of CDI were identified. Following the preventive use of LP299v, a notable reduction of the incidence rate of CDI was observed, from 12.1 to 1.1 per 1000 patients hospitalized in the Department of Nephrology, Transplantation and Internal Medicine, Medical University of Silesia in Katowice. The decrease in the incidence of CDI in the second period compared to the first period was highly significant (0.11% *vs.* 1.21% respectively; *p* = 0.0001) (Figure 1).

Nosocomial infection was identified in 14 patients (61% of total CDI cases) and community-acquired CDI was recognized in 3 (13%) patients. In six cases (26%), the infection was associated with the other health care facility and the onset of symptoms was suspected before the admission to our hospital.

The average time between the onset of symptoms and the diagnosis of CDI was 7.3 ± 7.0 days. The main clinical symptom of CDI observed in 23 (100%) patients was diarrhea with different intensity, sustained for 14.0 ± 10.5 days. The average number of stools per day was 6.2 ± 2.9. During the infection there was a reduction in the body weight in 12 patients. In these patients, the mean reduction of body weight was 5.9 ± 4.6 kg.

Abdominal pain, vomiting and fever were other frequent accompanying symptoms that occurred in 13 (56.5%), 3 (13.0%) and 19 (82.6%) patients, respectively.

From patients with clinical suspicion of CDI cases, the presence of toxins in the stool samples was detected in 22 using the enzyme immunoassay test. In the other remaining case, with an initial negative toxin assay result, the toxigenic culture test was positive.

Leukocytosis (>10 G/L) developed in 16 (69.6%) patients and leukopenia (<4 G/L) was observed in 1 (4.3%) case. Most of the CDI patients (91.3%) had elevated serum concentration of C-reactive protein (CRP). The average leukocytes count and CRP serum concentration in the study group was 18.8 ± 16.0 G/L and 89.3 ± 80.2 mg/L, respectively. A decreased hemoglobin concentration was found in 13 (56.5%) patients (by an average of 1.8 ± 1.7 g/dL) and hypokalemia (serum potassium concentration <3.5 mmol/L) was present in 6 (26.1%) patients.

Five (22%) patients developed severe infection and one of them died within 30 days after the onset of CDI symptoms (Table 2).

**Table 2:** Severe diseases associated with *Clostridium difficile* (*n* = 5).

	Before LP299v Use	After LP299v Use
Admission to hospital for treatment of community-acquired CDI (*n*)	3	0
Megacolon toxicum (*n*)	0	0
Colectomy (*n*)	0	0
Transfer to the intensive care unit due to CDI complications (*n*)	1	0
Death (*n*)	1	0

Three cases of a severe form of *C. difficile* infection were diagnosed in patients after solid organ transplantation and there was no case of severe CDI during the year of LP299v prophylaxis. The analysis of already known risk factors of severe CDI is shown in Table 3.

**Table 3:** Risk factors of severe course of CDI in the study group.

	Before LP299v Use (*n*/%)	After LP299v Use (*n*/%)
Age > 70 years	4/19	0/0
WBC > 20 G/L	7/33	1/50
Serum creatinine > 2mg/dL	16/76	0/0
Albumin < 2.5 g/dL	9/43	1/50
Bowel obstruction	0/0	0/0
Changes in the large intestine detected in CT	1/5	1/50
History of surgery in the last 30 days	4/19	1/50

During these two twelve-month periods of observation, three patients suffered from CDI recurrence, including two episodes after starting the *Lactobacillus plantarum 299v* as a routine prophylaxis. In these two cases, the recurrence of gastrointestinal symptoms and the severity of the infection (duration of diarrhea, number of stools per a day, average CRP serum concentrations) during LP299v prophylaxis was milder when compared with these same parameters before such a prophylaxis was introduced (Table 4).

**Table 4:** During the year before the introduction of LP299v CDI was diagnosed in 21 patients. In two of them, the CDI recurrence was diagnosed after starting the LP299v prophylaxis. The analysis of the clinical features of the recurrence of CDI in two patients, in whom CDI was diagnosed, before and after initiation of *Lactobacillus plantarum 299v* prophylaxis, were presented.

	Before LP299v Use	After LP299v Use
Duration of diarrhea (days)	28	9.5
Number of stools per a day	8	7
Abdominal pain (*n*)	2	2
Vomiting (*n*)	0	0
Fever (*n*)	2	2
Average leukocytes count (G/L)	14.1	14.4
Average CRP serum concentrations (mg/L)	96.5	43.8

Of the CDI patients, 56.5% were males. The mean age of CDI patients was 56 ± 14 years. CDI occurred more frequently in patients with advanced age. This disease was found in patients aged below 40, between 40 and 50 and above 50 years of age in three (13.0%), five (21.7%) and 15 (65.2%) patients, respectively.

**Figure 2:**
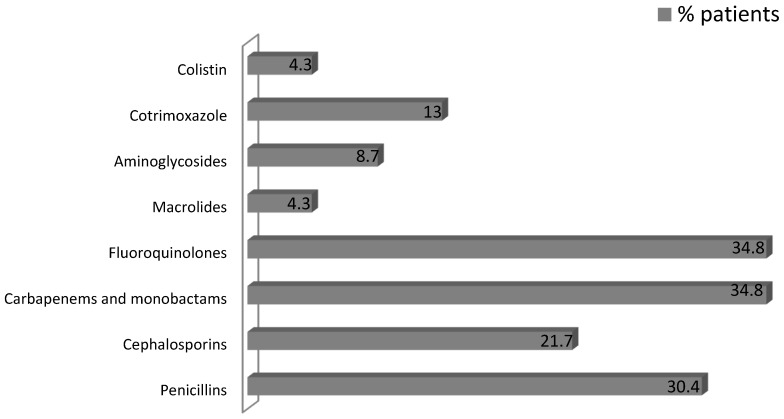
The antibiotic exposure in the CDI patients.

As a risk factor, 18 (78.3%) of patients had a history of antibiotic use in the previous four weeks before the onset of CDI symptoms and 11 patients (47.8%) were treated with more than one antibiotic. The clinical presentation of CDI was observed an average of 6.8 ± 5.2 days after the beginning of the antibiotic therapy. The most common indication for antibiotic use among CDI patients was urinary tract infection and fluoroquinolones, carbapenems and monobactams were the most frequently used drugs. The indications for antibiotic use among CDI patients are presented in Table 5 and the antibiotic exposure in the CDI group is shown in Figure 2.

**Table 5:** Antibiotic use indications among CDI patients.

The Indications for Antibiotic Use	*n*
Urinary tract infection	13
Pneumonia	1
Upper respiratory tract infection	1
Peritoneal dialysis-related infection	2
Other	1

In this observational, retrospective study, we also compared the use of antibiotics in the Department of Nephrology, Transplantation and Internal Medicine, Medical University of Silesia in Katowice, Poland in both periods of observation, before (October 2012 to October 2013) and after initiation of the use of LP299v (December 2013 to December 2014). The trend towards using less antibiotics (evaluated as a number of used antibiotic packages) in the second period compared to the first period was observed (2643 packages *vs.* 3481 packages). The use of antibiotics in both periods of observation is shown in Table 6.

**Table 6:** Number of used antibiotic packages in the Department of Nephrology, Transplantation and Internal Medicine Medical University of Silesia in Katowice, Poland before (October 2012 to October 2013) and after initiation of LP299v prophylaxis (December 2013 to December 2014).

	Number of Used Antibiotic Packages in the Period	
	Between October 2012 and October 2013	Between December 2013 and December 2014
Cephalosporins	1851	1228
Penicilins	390	353
Carbapenems and monobactams	196	126
Fluoroquinolones	304	300
Aminoglycosides	36	150
Tetracyclines	25	10
Tigecycline	3	19
Macrolides	32	7
Clindamycin	13	8
Cotrimoxazole	86	20
Glycopeptides	148	233
Linezolid	1	6
Colistin	3	3
Other	393	180

Proton pump inhibitors were used in 20 (86.9%) patients with CDI. None of them were treated with histamine 2 receptor blockers. The trend towards using less proton pump inhibitors (evaluated as the number of used packages) in the second period compared to the first period was observed (1085 packages *vs.* 1639 packages). In addition, 73.9% of CDI patients were hospitalized in our Department or in other hospitals during the three months before the onset of CDI symptoms and every third patient was hospitalized more than once.

## 4. Discussion

This study is so far one of the few which evaluated the effect of administration of *Lactobacillus plantarum 299v* in the prevention of *C. difficile* infection and the first one performed among patients hospitalized in a transplantation unit. The results obtained in our study have shown a significant decrease of the incidence of CDI after initiation of LP299v prophylaxis. Moreover, there was no case of severe CDI during the year of LP299v use. After beginning LP299v administration, CDI was diagnosed in two patients and, in both cases, it was defined as the recurrence of the infection. Nevertheless, the severity of the recurrent infection during intake of LP299v was milder.

Klarin *et al.* [33] investigated the impact of LP299v on *C. difficile* colonisation among 44 patients treated with antibiotics in the intensive care unit. None of the 22 critically ill patients who were given LP299v had detectable *C. difficile* toxins in the faecal samples, while colonisation with this pathogen was reported in 19% of the placebo group (*p* < 0.05). These findings suggest that the administration of LP299v during antibiotic treatment to patients, who are at high risk of CDI, is effective in the prevention of *C. difficile* colonisation. The authors did not evaluate the association between the incidence of CDI and LP299v use. Due to the retrospective nature of the current study, the analysis of *Clostridium difficile* colonisation during LP299v use was not possible.

A significant clinical and economic problem is CDI recurrence that may be diagnosed in up to 20% of CDI patients [7]. A small randomized, placebo-controlled study (*n* = 21) was performed to evaluate to efficacy of LP299v in the prevention of CDI recurrence. We observed a tendency to a lower incidence of recurrences during three months of observation in patients who received during the treatment metronidazole in combination with LP299v than in control group (metronidazole in combination with placebo) [32]. The follow-up of both patients who had suffered from recurrence of CDI after initiation of LP299v prophylaxis in our Department did not reveal another episode of this infection (data not shown).

Johansson *et al.* have documented a relationship between the intake of LP299v and increased concentration of short-chain fatty acids (SCFAs) in faecal samples from healthy volunteers [36]. This observation, in respect to the evidence of a positive correlation between the production of SCFAs, absorption of sodium and water in the colon, and reduction of diarrheal symptoms [37] led to further studies, including also patients with CDI. Wullt *et al.* performed an analysis, to explore whether the intake of LP299v affected the concentration of SCFAs in stool samples collected from 19 patients with the recurrence if CDI during and after treatment with metronidazole. These authors found that the administration of LP299v may reduce the negative influence of antibiotics on the fermentation process in the colon [32].

In a double blind, placebo controlled study Lönnermark *et al.* [27] showed, that the intake of *Lactobacillus plantarum 299v* in a dose 10 × 10^9^ CFU/day did not affect the occurrence of antibiotic associated diarrhoea. However, the authors reported a reduced incidence of gastrointestinal symptoms associated with antibiotic intake. In their observation, the risk of developing loose or watery stools and nausea was significantly lower among patients receiving LP299v. This effect was most significant during antibiotic treatment. Similarly, we observed the shorter duration of diarrhoea and decreased number of stools as well as lower serum C-reactive protein (CRP) concentrations in patients with CDI during LP299v prophylaxis. *C. difficile* toxins in fecal samples were found only in few patients enrolled into the study conducted by Lönnermark *et al.* [27]; nevertheless, there was no difference between the treatment and placebo groups in terms of *C. difficile* colonisation.

This study has some limitations, which have to be pointed out. The retrospective nature of the study and a low number of CDI patients do not allow us to make an unquestionable conclusion concerning effectiveness of routine administration of LP299v in the prevention of CDI. We could not exclude the effects of decreased use of antibiotics and proton pomp inhibitors on the prevalence of CDI in the second period of observation. The analysis of the number of used antibiotic packages also revealed changes in the main types of antibiotics given in our department. In the second period of observation, we noted less frequent use of cephalosporins and an increase of antibiotics use which have less effect on the risk of CDI, like aminoglycosides.

In conclusion, the results of our clinical, retrospective, single-center study indicate that routine administration of *Lactobacillus plantarum 299v* during treatment with antibiotics may prevent *C. difficile* infection, particularly in patients at high risk of CDI. With respect to promising results of this study, further large, randomized, clinical studies should be performed in order to prove the efficacy of LP299v in the prevention of CDI.
